# Mitrofanoff Appendicovesicostomy With Boari Flap for Complete Female Urethral Transection: A Case Report

**DOI:** 10.1002/iju5.70154

**Published:** 2026-02-17

**Authors:** Kohei Mori, Takehiro Iwata, Tatsushi Kawada, Takuya Sadahira, Yusuke Tominaga, Satoshi Katayama, Shingo Nishimura, Kensuke Bekku, Yuichiro Yamasaki, Motoo Araki

**Affiliations:** ^1^ Department of Urology Okayama University Graduate School of Medicine, Dentistry and Pharmaceutical Sciences Okayama Japan; ^2^ Department of Urology Kanagawa Children's Medical Center Yokohama Kanagawa Japan

**Keywords:** Boari flap, female urethral transection, Mitrofanoff

## Abstract

**Introduction:**

Female urethral complete transection caused by pelvic trauma is extremely rare, and no standard management has been established when urethral reconstruction is not feasible.

**Case Presentation:**

A woman in her twenties sustained an open pelvic fracture with perineal injury due to a traffic accident. Complete urethral transection was identified, and a suprapubic cystostomy was placed. After staged vaginal reconstruction and bladder function evaluation, a Mitrofanoff appendicovesicostomy was performed. Because the appendix was not enough to reach the umbilicus, a Boari flap was created to compensate for the length. Urodynamic evaluation showed improvement from a preoperative high‐pressure bladder to increased compliance postoperatively, though pharmacological management was still required. Postoperatively, the patient achieved stable clean intermittent catheterization without complications.

**Conclusion:**

The Mitrofanoff procedure can be an effective option in female urethral injuries where reconstruction is impossible. The addition of a Boari flap may expand its applicability by overcoming conduit length limitations.

**Keynote Message**

For female patient, in urinary diversion procedures, appearance is a key consideration alongside functionality. The Mitrofanoff procedure can be an effective option in female urethral injuries where reconstruction is impossible. The urodynamic study enables the implementation of appropriate drug therapy.

## 1 Introduction

Female urethral injuries are rare, accounting for only 1%–2% of pelvic fracture urethral injuries in women and children [1]. Complete transection is especially challenging, as conventional urethral reconstruction requires extensive mobilization with a risk of urethrovaginal fistula and incontinence [2]. When primary repair is not feasible, urinary diversion with a continent catheterizable channel becomes an important alternative.

The Mitrofanoff procedure, which uses the appendix to create a catheterizable stoma, was originally described for pediatric neurogenic bladder but has since been applied to adult trauma cases [3]. Here we present a rare case of adult female complete urethral transection successfully managed by Mitrofanoff appendicovesicostomy with Boari flap.

## 2 Case Presentation

A woman in her twenties was brought to the emergency department following a forklift accident. She sustained an open pelvic fracture, buttock laceration, and perineal injury. The external urethral meatus could not be identified, and suprapubic cystostomy was placed. Vaginal wall damage necessitated colostomy and perineal wound repair.

**FIGURE 1:**
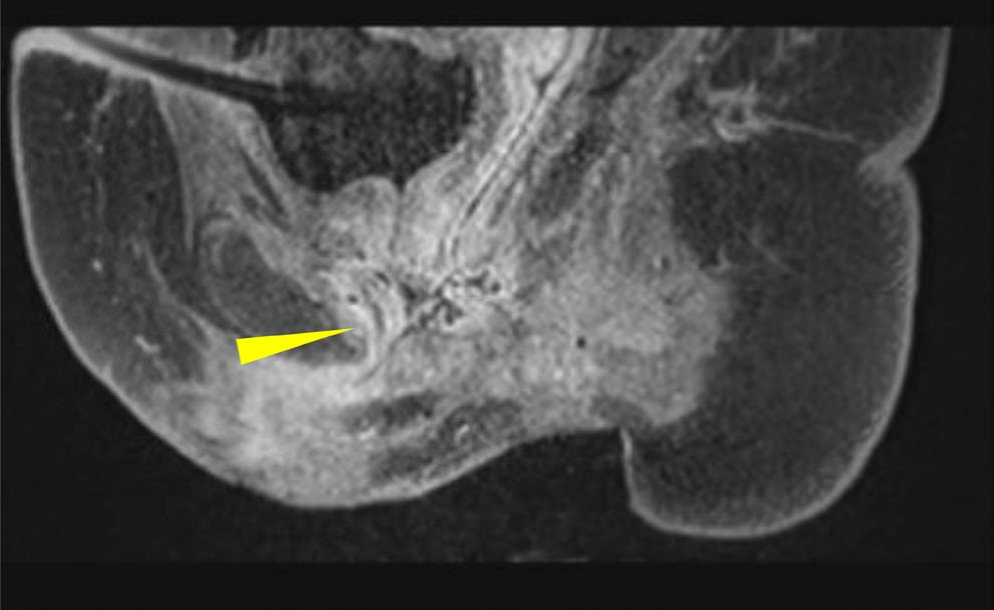
MRI image showing complete mid‐urethra transection and displacement (yellow arrow: Mid‐urethra transection).

At 11 months post‐injury, a pull‐through urethroplasty was attempted, but was abandoned due to complete urethral obliteration. Magnetic resonance imaging revealed mid‐urethral transection with ventral displacement of the proximal stump, confirming that reconstruction would be technically difficult (Figure 1). She was referred to our institution for further management.

### 2.1 Bladder Function Evaluation

At *X* + 3 years and 8 months after injury, urodynamic study revealed bladder capacity of 210 mL (detrusor pressure 14 cmH_2_O), with bladder compliance at 15.9 mL/cmH_2_O. These findings indicated a high‐pressure bladder. The administration of β3 agonist and anticholinergic was initiated, resulting in improvement of bladder capacity to 330 mL.

### 2.2 Surgical Procedure

**FIGURE 2:**
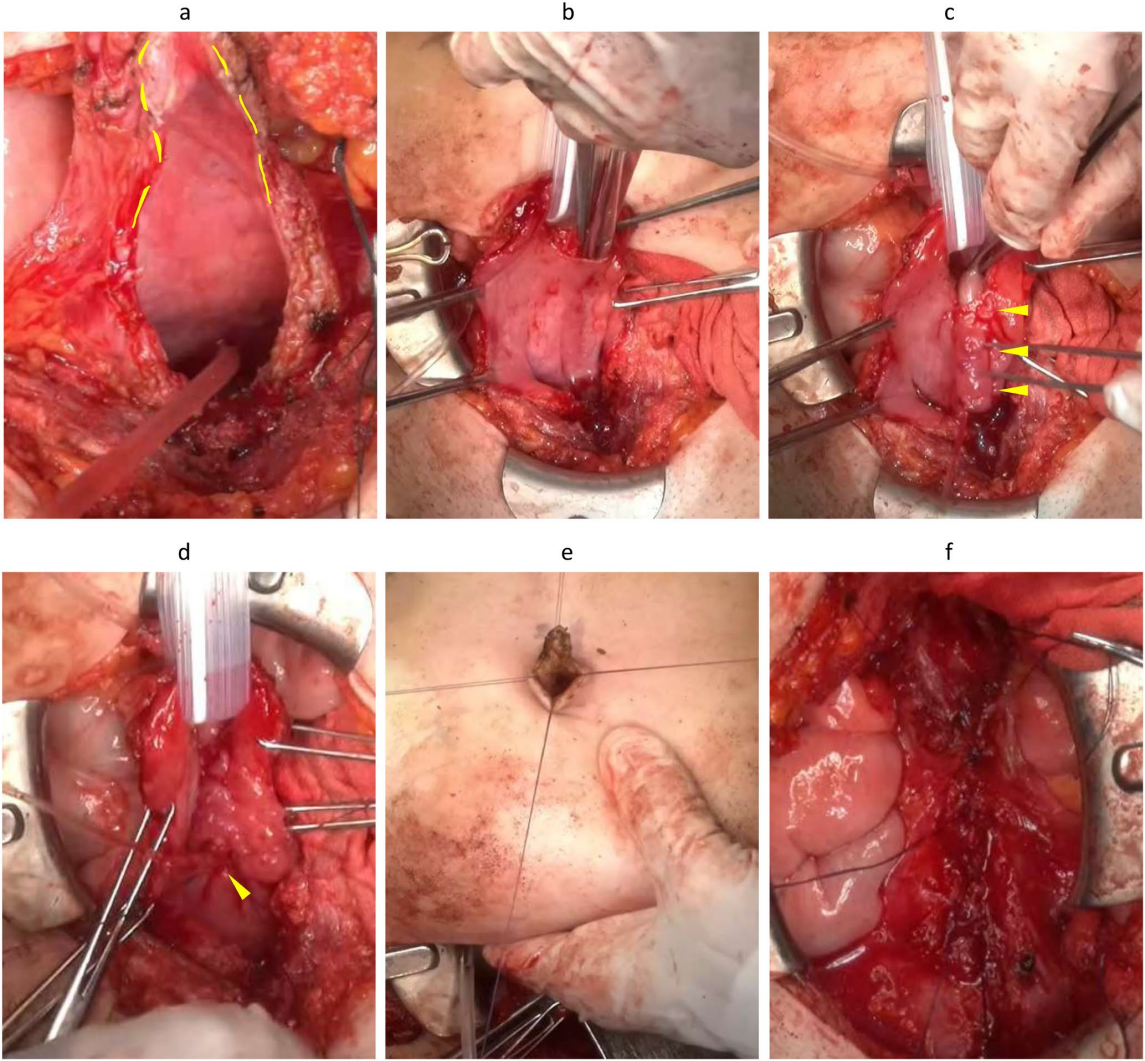
Procedure of the Mitrofanoff appendicovesicostomy combined with Boari flap. (a) U‐shaped incision of the anterior bladder wall (yellow dot line: Boari flap). (b) Submucosal tunnel 55 mm. (c) Pull the appendix into the submucosal tunnel (yellow arrow: Submucosal tunnel). (d) Intermittent suture of the bladder mucosa with the appendix mucosa (yellow arrow: Appendix mucosa). (e) V flap incision of the umbilicus and intermittent suture with the appendix mucosa. (f) Closure of the anterior bladder wall.

An open Mitrofanoff appendicovesicostomy was performed. The length of appendix was not enough to fix the umbilicus, so a Boari flap was fashioned from the anterior bladder wall to extend conduit length. The appendix was tunneled submucosally into the bladder and exteriorized at the umbilicus (Figure 2).

### 2.3 Postoperative Course

**FIGURE 3:**
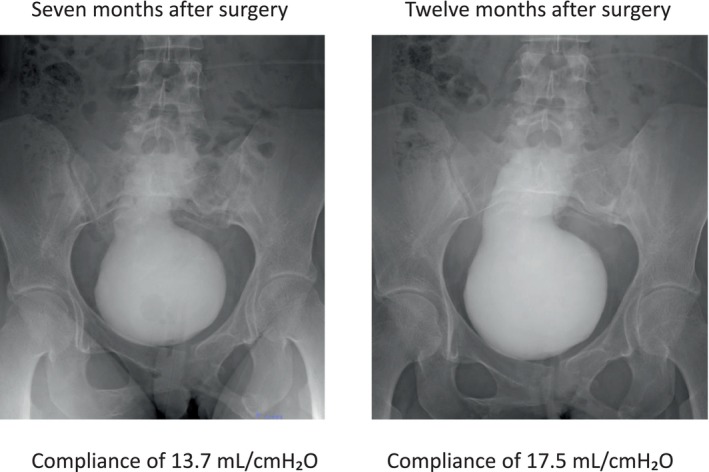
Abdomen and Cystography at 7 months after the operation in Videourodynamics study.

The umbilical catheter was removed at 4 weeks after the Mitrofanoff operation, confirming that clean intermittent catheterization (CIC) could be performed. At 3 months after the operation, a β3 agonist and an anticholinergic were discontinued. The ultrasound findings showed absence of hydronephrosis, and the patient's course was favorable. At 7 months after the operation, Videourodynamics study demonstrated that bladder capacity at 261 mL (detrusor pressure 19 cmH_2_O), with compliance of 13.7 mL/cmH_2_O (Figure 3). Bladder compliance remained low after surgery, indicating necessity of oral medication. Therefore, the patient was prescribed an anticholinergic drug. A follow‐up video urodynamic evaluation was performed at 12 months after the operation, revealing improvement of bladder capacity at 297 mL (detrusor pressure 17 cmH_2_O), with compliance of 17.5 mL/cmH_2_O. The patient continues CIC six times daily with stable renal function and absence of hydronephrosis.

## 3 Discussion

**FIGURE 4:**
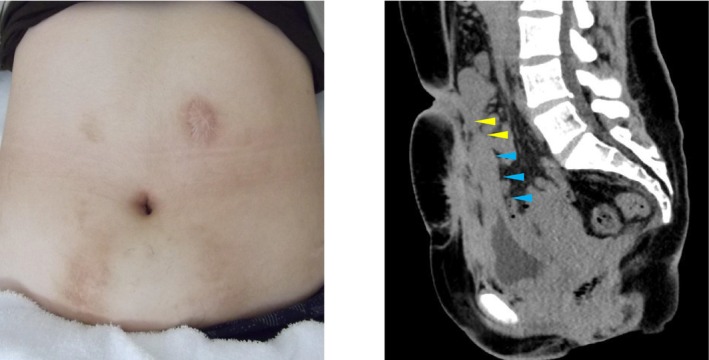
Appearance of the catheterizable channel (yellow allow: Appendix conduit, blue allow: Boari flap).

Female urethral injury is extremely rare, with complete transection representing one of the most challenging scenarios in reconstructive urology [2]. Conventional reconstruction requires extensive mobilization of periurethral tissue, which has an increased risk of fistula and incontinence. When reconstruction is not feasible, continent urinary diversions provide important alternatives. Given the patient's young age and the need for a long‐term catheterizable channel, we had to consider both functional and cosmetic outcomes in planning surgery. In particular, umbilical stoma via Mitrofanoff appendicovesicostomy was favored for its discreet and aesthetically acceptable appearance, which is especially important in young female patients (Figure 4).

Our case highlights two additional aspects: bladder physiology and technical modification. Preoperative urodynamics revealed a high‐pressure bladder with low compliance (15.9 mL/cmH_2_O), which can cause vesicoureteral reflux and renal dysfunction. We define that low bladder compliance is less than 10–20 mL/cmH_2_O, a clinical threshold widely recognized as a risk factor for renal dysfunction [4]. Optimization with medications improved bladder capacity and compliance before surgery. However, postoperative evaluation at 7 months still demonstrated low compliance (13.7 mL/cmH_2_O), indicating oral medications. At the 12 months follow‐up video urodynamics, bladder compliance (17.5 mL/cmH_2_O) had been improved with the anticholinergic therapy. This emphasizes that long‐term bladder surveillance remains mandatory even after successful continent diversion in order to preserve renal function.

The Mitrofanoff procedure is widely established as a reliable technique in children with neurogenic bladder. Crucially, this surgery allows patients to maintain urinary function, significantly improving their quality of life. The Mitrofanoff procedure has been reported to have low complication rates such as stricture or incontinence, and to yield excellent long‐term outcomes. On the other hand, when the appendix cannot be used, the Monti procedure using ileum was also well established. While both procedures are effective in preserving urinary function, some reports indicate that the Monti procedure has a high complication rate and reoperation rate [5]. This is attributed to the increased susceptibility to conduit ischemia and kinking due to the tubular structure of the ileum. Furthermore, Polm et al. reported a long median follow‐up of 12.4 years; a key feature of their study was the comparison of reconstructive techniques using tubularized bladder flap (TBF) [6]. They demonstrated that reoperation was more common in Monti procedure compared with Mitrofanoff and TBF. The reason was a higher rate of incontinence in the Monti group compared to others.

Adult cases present unique challenges because the distance between the bladder and the umbilicus is longer than in pediatric patients. In our case, the patient was a young female who prioritized cosmetic outcomes. Therefore, we selected a urinary diversion to the umbilicus, which offers superior cosmetic results compared to the lower abdominal wall. Consequently, the submucosal tunnel shortened due to reduced bladder capacity, we considered this might increase the risk of incontinence. To mitigate this, we combined Boari flap with Mitrofanoff appendicovesicostomy for ensuring the length of the submucosal tunnel (Figure 3). To our knowledge, this is the first reported case of combining a Boari flap with a Mitrofanoff appendicovesicostomy. This technique avoids the need for bowel segments and provides a cosmetically favorable umbilical stoma, which is often important for patients.

There are some limitations to this report. As this is a single case study with a follow‐up period of 12 months, the long‐term durability of combining a Boari flap with a Mitrofanoff appendicovesicostomy remains to be fully established. Continued, rigorous surveillance is mandatory to monitor for potential late‐onset complications, including stomal stenosis, incontinence, or renal dysfunction.

In summary, the Mitrofanoff procedure remains a feasible alternative in female complete urethral transection, provided that bladder function is optimized preoperatively and closely monitored thereafter. The combination with a Boari flap expands its applicability in adults with insufficient appendix length.

## 4 Conclusion

The Mitrofanoff procedure is a reliable alternative for female patients with complete urethral transection when reconstruction is impossible. Careful preoperative evaluation and long‐term follow‐up are essential.

## Ethics Statement

The authors have nothing to report.

## Consent

The authors have nothing to report.

## Conflicts of Interest

The authors declare no conflicts of interest.

## Data Availability

The data that support the findings of this study are available on request from the corresponding author. The data are not publicly available due to privacy or ethical restrictions.
